# Evaluating the Surface Topography of Pyrolytic Carbon Finger Prostheses through Measurement of Various Roughness Parameters

**DOI:** 10.3390/jfb7020009

**Published:** 2016-04-14

**Authors:** Andrew Naylor, Sumedh C. Talwalkar, Ian A. Trail, Thomas J. Joyce

**Affiliations:** 1 School of Mechanical and Systems Engineering, Newcastle University, Newcastle upon Tyne, England NE1 7RU, UK; thomas.joyce@newcastle.ac.uk; 2 Upper Limb Research Unit, Wrightington Hospital, Wigan, England WN6 9EP, UK; sctalwalkar@gmail.com (S.C.T.); ian.trail@wwl.nhs.uk (I.A.T.)

**Keywords:** roughness parameters, surface topography, skewness, kurtosis, pyrolytic carbon, proximal interphalangeal joint

## Abstract

The articulating surfaces of four different sizes of unused pyrolytic carbon proximal interphalangeal prostheses (PIP) were evaluated though measuring several topographical parameters using a white light interferometer: average roughness (*S_a_*); root mean-square roughness (*S_q_*); skewness (*S_sk_*); and kurtosis (*S_ku_*). The radii of the articulating surfaces were measured using a coordinate measuring machine, and were found to be: 2.5, 3.3, 4.2 and 4.7 mm for proximal, and 4.0, 5.1, 5.6 and 6.3 mm for medial components. ANOVA was used to assess the relationship between the component radii and each roughness parameter. *S_a_*, *S_q_* and *S_sk_* correlated negatively with radius (*p* = 0.001, 0.001, 0.023), whilst *S_ku_* correlated positively with radius (*p* = 0.03). Ergo, the surfaces with the largest radii possessed the better topographical characteristics: low roughness, negative skewness, high kurtosis. Conversely, the surfaces with the smallest radii had poorer topographical characteristics.

## 1. Introduction

**Figure 1:**
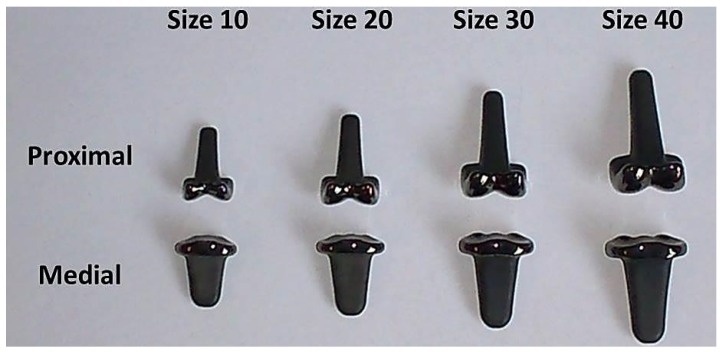
The relative sizes of the pyrolytic carbon prostheses evaluated in the study.

The pyrolytic carbon proximal interphalangeal prosthesis (PIP) is a two part prostheses designed to mimic the natural bicondylar anatomy of the PIP finger joint. This prosthesis consists of both proximal and medial components and comes in four nominal sizes: size 10; size 20; size 30 and size 40 (Figure 1). The pyrolytic carbon PIP has yielded mixed clinical results [1]. Clinical studies conducted by Wijk *et al.* [2] and Chung *et al.* [3] both show the prosthesis in a positive light, with the relatively low complication rates of 0.14 (95% CI 0.07–0.26) and 0.23 (95% CI 0.08–0.5), respectively. Conversely, studies carried out by Herren *et al.* [4] and Nunley *et al.* [5] both show the prosthesis in a less positive light, with high complication rates of 0.72 (95% CI 0.49 to 0.88) and 0.71 (95% CI 0.36–0.92), respectively. Not all of the complications can be explained clinically, and the underlying causes for complications and revisions are not fully understood.

In an attempt to shed further light on the clinical studies, both *ex vivo* [6] and *in vitro* studies [7] have been conducted. The *ex vivo* study [6] evaluated a range of explanted pyrolytic carbon prostheses from the hand, finding the largest component (carpometacarpal) to yield the lowest roughness (12 nm). Conversely, the smallest of the prostheses evaluated (distal interphalangeal) yielded the highest average roughness values: with a combined mean of 37 nm and a range of 17 nm to 66 nm. The *in vitro* study [7] evaluated the two largest available sizes of pyrolytic carbon proximal interphalangeal prostheses (size 30 and size 40), tested to five million cycles of flexion-extension. All four of the larger components and three of the smaller components either showed no change or only moderately increased in roughness. The remaining smaller component reached more than double its original roughness value at five million cycles (from 37 to 75 nm).

When considering topographical parameters, both average roughness and root mean-square roughness have been utilized in previous studies to evaluate the quality of joint prosthesis articulating surfaces [6,7,8,9,10,11,12,13,14]. These parameters quantify the relative heights of asperities across the surface; however, neither of them are sensitive to small changes in profile [15,16].

Although average roughness (*S_a_*) and root mean-square roughness (*S_q_*) have both been calculated from the topographical plots detailed in this manuscript, two additional parameters have been calculated: skewness (*S_sk_*) and kurtosis (*S_ku_*). *S_sk_* is a measure of height distribution about the profile line: positive *S_sk_* indicates the presence or more peaks; negative *S_sk_* indicates the presence of more valleys, and *S_sk_* ≈ 0 indicates symmetrical height distribution about the profile line. *S_ku_* is a measure of how densely or sparsely peaks and valleys are distributed across the measured surface. Methods of calculation and further description of these parameters are covered in the experimental section of this manuscript. A previous tribological study with an industrial emphasis [16] has documented that all four of these topographical parameters have been obtained simultaneously from different grades of 100Cr6 steel samples. To the knowledge of the authors, this present study is the first of its kind to consider all four of these parameters concurrently, from the same topographical plots, to evaluate the articulating surfaces of joint prostheses.

The purpose of this present study is to identify any relationships between measured radii of the unused prosthesis components and the topographical properties measured from the articulating surfaces. Historically, only one or two topographical parameters have been used to evaluate the surfaces of artificial joints [6,7,8,9,10,11,12,13,14]. In this present study, a total of four parameters have been used: *S_a_*; *S_q_*; *S_sk_*; and *S_ku_*.

## 2. Results and Discussion

### 2.1. Topographical Results

The size 10 and 20 proximal components exhibited mean *S_a_* values of: 69.2 nm (95% CI 63.4–74.8 nm) and 68.1 nm (95% CI 62.1–74 nm), respectively (Table 1), both significantly higher than the 50 nm threshold dictated by ISO 7206-2 [17] (*p* < 0.05). Conversely, the size 30 and 40 proximal components exhibited mean *S_a_* values of: 36.8 nm (95% CI 30.8–41.2 nm), and 25.9 nm (95% CI 23.6–28.3 nm), respectively, both significantly lower than the 50 nm threshold (*p* < 0.05). The mean *S_q_* values were higher than the *S_a_* values. The size 10 and 20 proximal components once again yielded results significantly higher than the 50 nm threshold (*p* < 0.05), with values of: 88 nm (95% CI 80.7–95.3 nm) and 87.9 nm (95% CI 80.5–95.2 nm), respectively. For the size 40 proximal component, the mean *S_q_* value also reflected the *S_a_* result, with a mean of 33.7 nm (95% CI 30.1–36.5 nm), significantly lower than the 50 nm threshold (*p* < 0.05). The size 30 proximal component, however, yielded a mean *S_q_* of 46.7 nm (95% CI 40.2–53.2 nm), close enough to the 50 nm threshold to accept the null hypothesis (*p* > 0.05). Mean *S_a_* and *S_q_* values for the medial components all fell below 50 nm. However, the size 10 and 20 medial components had mean *S_q_* values of: 46.4 nm (95% CI 34–58.7 nm), and 47.1 nm (95% CI 42.6–51.6 nm), respectively. These are close enough to 50 nm to accept the null hypothesis (*p* > 0.05).

The majority of the *S_sk_* results fell within the recommended guidelines [18], specifying a skewness of no less than −1.5 and no greater than +1.5. The midpoint of this range was used as null hypothesis (*S_sk_* ≈ 0) and was subsequently accepted in all but two instances (*p* > 0.05). The two instances where the null hypothesis was rejected (*p* < 0.05) was the size 40 proximal and medial components, which yielded *S_sk_* values of: −0.43 (95% CI −0.685 to −0.175); and −0.62 (95% CI −1.08 to −0.161) respectively. For all of the proximal and medial components, *S_ku_* significantly exceeded the recommended value of 3 [15] (*p* < 0.05). The size 40 proximal and medial components yielded considerably higher *S_sk_* values than the other proximal and medial components, with mean values of: 11.4 (95% CI 7.84–14.97); and 29.74 (95% CI 15.22 to 44.26) respectively. Furthermore, the medial components yielded higher *S_sk_* values than the proximal components.

**Figure 2:**
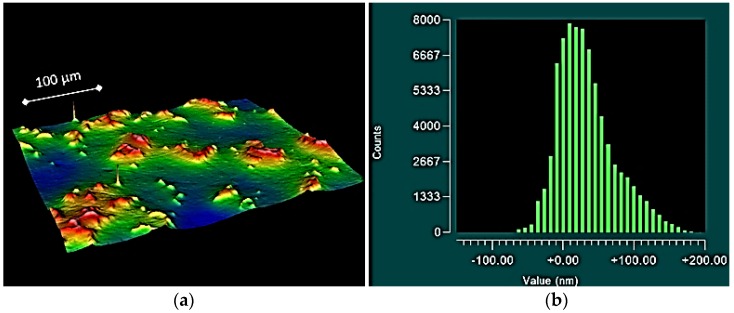
Topographical plot showing a surface with peaks (**a**) and histogram exhibiting a positively skewed distribution (**b**), obtained from medial component size 10.

**Figure 3:**
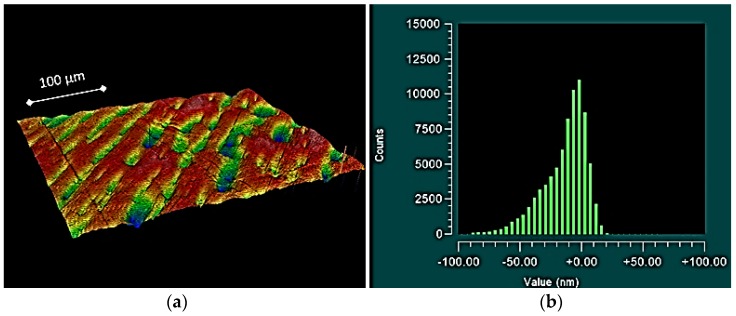
Topographical plot showing a surface with valleys (**a**) and histogram exhibiting a negatively skewed distribution (**b**), obtained from proximal component size 40.

**Figure 4:**
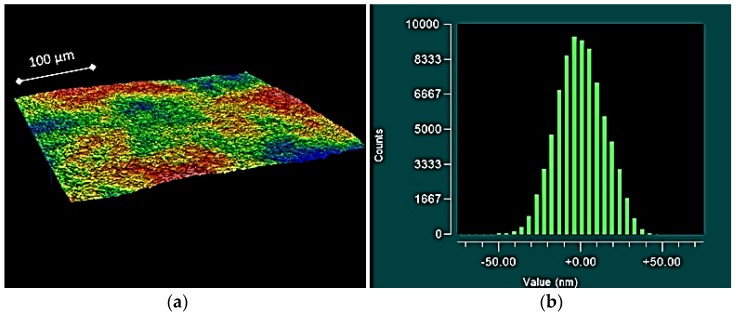
Topographical plot showing a flat surface (**a**) and histogram exhibiting a uniform Gaussian distribution (**b**), obtained from medial component size 30.

To add further context to these results, the topographical plots of the smaller components often exhibited raised asperities (Figure 2), resulting in: the higher observed roughness (both *S_a_* and *S_q_*); and the higher observed *S_sk_* values. For the larger components, the topographical plots often exhibited valleys along the surface (Figure 3), providing an explanation for the low negative *S_sk_* values and high *S_ku_* values. In some cases, for the mid-size prostheses (size 20 and size 30); symmetrically distributed surfaces exhibiting a uniform Gaussian distribution were observed (Figure 4).

### 2.2. Linear Regression and ANOVA

**Figure 5:**
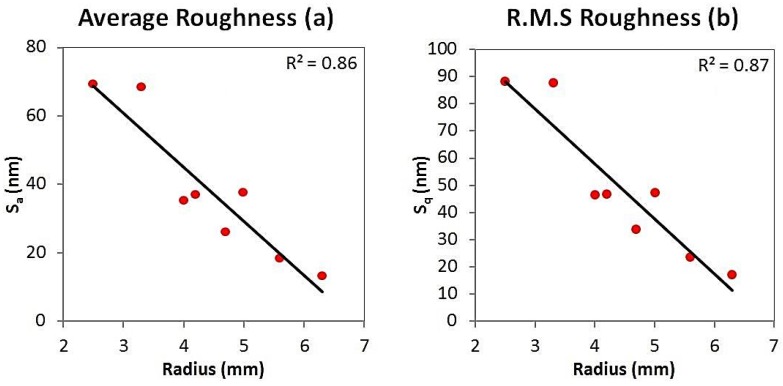
Average roughness linear regression model (**a**) and root mean-square linear regression model (**b**).

**Figure 6:**
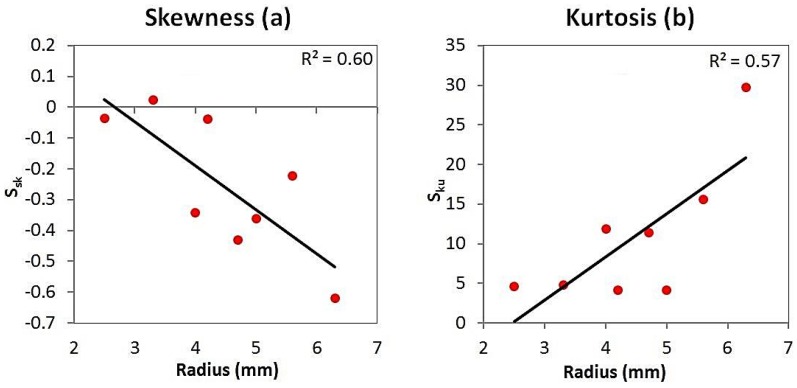
Skewness linear regression model (**a**) and kurtosis linear regression model (**b**).

The linear regression models demonstrated similar strong relationships for *S_a_* and *S_q_* with respect to the measured radii of the articulating surfaces. The average roughness model (Figure 5a) exhibited a negative correlation between *S_a_* and radius, with a *R*^2^ value of 0.86. Similarly, the root mean-square roughness model (Figure 5b) also exhibited a negative correlation between *S_q_* and radius, with a *R*^2^ value of 0.87. Furthermore, ANOVA (Table 2) showed these relationships to have a high significance (*p* = 0.001 for both models). The skewness model (Figure 6a) also exhibited a negative correlation between *S_sk_* and radius. Although this model had a weaker fit, with an *R*^2^ value of 0.6, the trend was still significant (*p* = 0.023). The kurtosis model (Figure 6b) exhibited a positive correlation between *S_ku_* and radius. As with the skewness model, the fit was also weaker, with a *R*^2^ value of 0.57, yet the trend still had a high level of significance (*p* = 0.03).

### 2.3. Prior Assessment of Joint Prostheses of the Hand

In order to critically evaluate the methods and results presented in this article, one must look to other relevant studies that have analysed the surfaces of joint prostheses. When considering two-piece implants of the hand, surface metrology has previously been used to evaluate metal on polymer finger prostheses subjected to *in vitro* wear tests [10,12]. One *in vitro* study evaluating a stainless steel (SS) on ultra-high molecular weight polyethylene (UHMWPE) metacarpophalangeal prostheses [12] utilised both 2D and 3D techniques. After evaluating the SS proximal component post wear test, it was found to have increased in 3D average roughness from 0.147 to 0.209 µm, measured using a ZYGO white light interferometer (Zygo Corporation, Middlefield, Connecticut, United States). Conversely, the UHMWPE medial component was found to have a reduction of 1.03 µm in 2D roughness, measured using a 2D profilometer. Another *in vitro* study, evaluating cobalt-chromium (CoCr) on UHMWPE PIP prostheses [10], used a white light interferometer to measure 3D roughness. Upon completion of the test regime, the CoCr proximal component was found to have slightly increased in average roughness (6 nm), whilst the UHMWPE medial component had an overall reduction in average roughness (0.22 µm). Collected micrographs also provided evidence of polishing.

Focusing more specifically on pyrolytic carbon joints of the hand, an *in vitro* study evaluating PIP prosthesis [7] also utilised a white light interferometer to measure 3D roughness parameters. Two medial pyrolytic carbon components and one proximal pyrolytic carbon component exhibited a negligible change in average roughness post wear testing. However, one condyle of a size 30 proximal component exhibited a substantial increase in average roughness with a measurement of 42 nm prior to testing and a measurement of 122 nm after testing. Furthermore, the mean skewness decreased from −0.25 prior to testing to −1.4. These findings, combined with a distinct wear scar presented in a topographical plot demonstrated that material was progressively removed or displaced over the duration of the wear test. A separate *ex vivo* study [6] evaluated a range of explanted pyrolytic carbon prostheses from the hand. Only 3D average roughness was measured, with the largest prosthesis (carpometacarpal) yielding the lowest average roughness (12 nm). Conversely, the smallest of the prostheses evaluated (distal interphalangeal) yielded the highest average roughness values: with a combined mean of 37 nm; and a range of 17 to 66 nm. This is consistent with the findings documented in this article. It has been previously suggested that smaller components are more difficult to polish [19]; however, further investigation would be useful.

### 2.4. What Are the Most Appropriate Topographical Parameters?

A key point of discussion is the comparability of *S_a_* and *S_q_*. In this present study, both parameters exhibited similar trends with respect to prosthesis size/radius. The main difference is the higher magnitude attributed to the *S_q_* parameter, on account of it being a root mean-square calculation. Both 2D (*R_a_*) and 3D (*S_a_*) average roughness values are accepted to provide a good gauge for the variation in heights obtained from a topographical plot; however, these parameters are not sensitive to small changes in profile on the surfaces [15,16]. Both the 2D (*R_q_*) and 3D (*S_q_*) root mean-square values are more sensitive to variation in heights, providing larger estimates for roughness than either *R_a_* or *S_a_*. Root mean-square roughness is more sensitive to heights than average roughness, yet still does not provide a detailed description of the surface [15,16]. Furthermore, the standard threshold of 50 nm is recommended only for average roughness, with no mention of root mean-square roughness [17]. From this, it can be argued that *S_a_* is a more appropriate way of quantifying surface roughness than *S_q_*.

Aside from joints of the hand, prostheses of: shoulders [8]; knees [9]; and hips [11] have also been evaluated using a white light interferometer. As these prostheses are much larger than those of the hand, reliable comparisons cannot be drawn. It is, however, worth reporting the topographical parameters used in these studies. When considered along with the studies evaluating joint prostheses of the hand [6,7,10,12], it can be ascertained that: average roughness is the most frequently used parameter to assess the articulating surfaces of prostheses [6,7,9,10,11,12,13], with few studies having considered skewness [7,9,13], and two studies having considered root mean-square roughness [9,13]. Kurtosis has been overlooked altogether, which has made it challenging to interpret the *S_ku_* values documented in this article, with no comparative data available. All of the measured *S_ku_* values are significantly greater than the threshold of 3 (*p* < 0.05), which indicates that there are many sharp peaks or valleys formed on the surface. Furthermore, the kurtosis model (Figure 6b) indicated that *S_ku_* increased with prosthesis radius. It is also pertinent to mention that the *S_sk_* guidelines [18] (skewness of no less than −1.5 and no greater than +1.5), which was used to govern the null hypothesis for *S_sk_* *z*-tests (Table 1), was based on the assumption that too many peaks or valleys is detrimental to the surface, and that *S_sk_* = 0 ± 1.5 is the ideal. The null hypothesis was accepted for size 10; size 20; and size 30 measurements. The null hypothesis was rejected for the size 40 components on account of lower negative *S_sk_* values attributed to the components.

**Figure 7:**
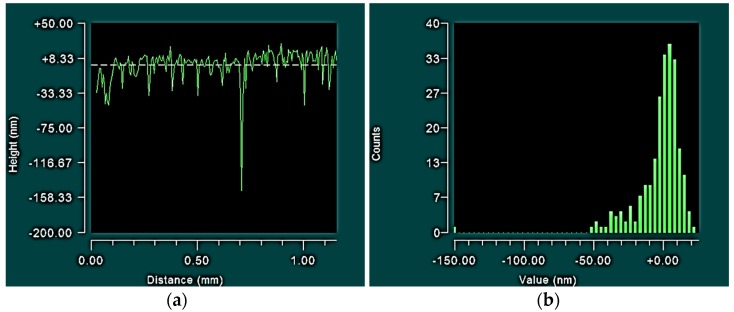
A 2D profile plot (**a**) and histogram (**b**) taken across a series of valleys demonstrating high 2D kurtosis (*R_ku_* = 27.69) and a negative skewness (*R_sk_* = −3.62).

A highly cited study which evaluated the roughness parameters of different grades of steel [16] actually indicates that a low negative skewness, combined with a high kurtosis, is advantageous for distributing lubrication. From a series of pin on disc tests, using synthetic oil as a lubricant, the sample with the lowest *S_sk_* (−3.11) and the highest *S_ku_* (23.2) yielded a very low coefficient of friction (0.1), which is indicative of a mixed of lubrication regime [20]. It has been proposed that the characteristic sharp valleys associated with high kurtosis and negative skewness (Figure 7) provide nanoscale reservoirs for lubricant [21]. It has also been noted that these reservoirs are too small to act as traps for typical wear particles ranging from 10 to 100 µm [16]. Hence, the combination of negative skewness and high kurtosis observed for the size 40 prosthesis (Figure 3) is advantageous.

## 3. Experimental Section

### 3.1. Roughness Parameters

A ZYGO NewView 5000 white light interferometer (Zygo Corporation, Middlefield, CT, USA) was used to obtain topographical plots at the articulating surfaces of the prostheses. These plots not only provided images, but also numerical values used to quantify the surfaces. Four salient topographical parameters were selected, with methods of calculation provided by the recognized standard for surface measurement, ISO 25178-2 [22]. Average roughness (*S_a_*) (Equation (1)) is a good gauge for the variation in heights about the profile line. However, two highly cited studies, Gadelmawla *et al.* [15] and Sedlaček *et al.* [16], have stated that average roughness is not sensitive to small changes in profile:

(1) $$S_{a}=\frac{1}{A}\iint_{A}|z(x,y)|dxdy$$

Both Gadelmawla *et al.* [15] and Sedlaček *et al.* [16] have stated that root mean-square roughness (*S_q_*) (Equation (3)) is more sensitive to variation in heights than average roughness, yet still does not provide a detailed description of the measured surface:

(2) $$S_{q}=\sqrt{\frac{1}{A}\iint_{A}z^{2}(x,y)dxdy}$$

The dimensionless value of skewness (*S_sk_*) (Equation (3)) was also obtained. Skewness provides a more detailed description of the measured surface than both average roughness and root mean-square roughness. It is described as being sensitive to sporadic deep valleys or high peaks [15,16]. Positive skewness indicates the presence or more peaks, negative skewness indicates the presence of more valleys, and zero skewness indicates symmetrical height distribution about the profile line. Further to this, Gadelmawla *et al.* [15] has stated that skewness can be used to distinguish between dissimilar surfaces that have the same average roughness or the same root mean-square roughness values:

(3) $$S_{sk}=\frac{1}{{S_{q}}^{3}}\left(\frac{1}{A}\iint_{A}z^{3}(x,y)dydx\right)$$

As with skewness, kurtosis (*S_ku_*) (Equation (4)) also provides a more detailed description of the measured surface than both average roughness and root mean-square roughness. Kurtosis describes the sharpness of the probability density of a surface [15,16]. If *S_ku_* < 3, then the surface has relatively few high peaks and few low valleys. Conversely, if *S_ku_* > 3, then the surface has relatively many high peaks and many low valleys:

(4) $$S_{ku}=\frac{1}{{S_{q}}^{4}}\left(\frac{1}{A}\iint_{A}z^{4}(x,y)dydx\right)$$

### 3.2. Samples

Four nominal sizes of prosthesis were selected for roughness evaluation: size 10; size 20; size 30; and size 40 (Figure 1). The radii of the proximal condyles and the medial plateaux were measured using a Mitutoyo Legex 322 coordinate measuring machine (CMM) (Mitutoyo Corporation, Kawasaki, Kanagawa, Japan). In ascending order of nominal size, the radii of the proximal condyles were: 2.53, 3.28, 4.15, and 4.7 mm, respectively. The corresponding radii of the medial plateaux were: 3.98, 5.08, 5.60, and 6.30 mm, respectively.

The measurement window for each topographical plot obtained was 317 µm × 238 µm, covering a spatial area of 75,446 µm^2^. Although it is possible to use larger measurement windows for flat surfaces, the small radii of the prostheses limited the surface area that could be successfully measured. To compensate for this, twenty topographical measurements were taken from each prosthesis pair using the white light interferometer, ten per proximal/medial component. For each condyle and plateau evaluated, five topographical plots were obtained: one measurement taken at the centre; then four peripheral measurements taken, equispaced at 90°. As there were two prostheses for each nominal size, 160 measurements were taken in total.

### 3.3. Statistical Methods

A one sample *z*-test was conducted to evaluate the data collected for each of the four roughness parameters. This was performed using MINITAB and was used to calculate the *z*-score, upper and lower confidence levels, and the *p*-value. The two-tailed normal approximation method was used with the null and alternative hypotheses expressed by means of the observed and hypothesized sample proportions. As ISO 7206-2 (the standard for metal and ceramic prostheses) dictates a threshold roughness of 50 nm [17], it was deemed prudent to use this as the hypothesised proportion for both *S_a_* and *S_q_*. For *S_sk_*, the hypothesised proportion was taken as the midpoint of −1.5 and +1.5 (zero), with care taken to note any outliers below −1.5 or above +1.5, as these scenarios indicate the presence of deep valleys and high peaks respectively [18]. For *S_ku_*, the hypothesised proportion was taken as 3—below this indicates the presence of relatively few high peaks and low valleys, above this indicates the presence of relatively many high peaks and low valleys [15,16]. Aside from sample statistics, linear regression and analysis of variance (ANOVA) were both used to determine the strength and validity of the relationships between the parameters and prosthesis radii.

## 4. Conclusions

The pyrolytic carbon prostheses with the largest radii have been shown to possess superior topographical properties, exhibiting: the lowest *S_a_* values; the lowest *S_q_* values; low negative *S_sk_* values; and high *S_ku_* values. Conversely, prostheses with the smallest radii have exhibited inferior topographical properties. *S_a_* and *S_q_* exhibited very similar trends with respect to prosthesis radius. It is proposed here that the use of only one of these two parameters would be sufficient to gauge 3D roughness. *S_a_* would appear to be the better of the two as it is the standard value used to assess the roughness of joint prostheses [17]. This study has established relationships between prosthesis size (radius) and topographical properties (*S_a_*, *S_q_*, *S_sk_*, *S_ku_*). It is clear that the smaller pyrolytic carbon components are rougher than the larger components.

## Figures and Tables

**Table 1:** Data summary from statistical hypothesis test displaying: 95% confidence intervals; *z*-scores; and *p*-values for the respective sample means of *S_a_*, *S_q_*, *S_sk_*, and *S_ku_*.

Parameter	Size	Proximal Component			Medial Component		
		Mean (95% CI)	*z*-Score	*p*-Value	Mean (95% CI)	*z*-Score	*p*-Value
*S_a_* (nm)	Size 10	69.2 (63.4–74.8)	6.52	0.00	34.9 (25.6–44.1)	−3.20	0.00
	Size 20	68.1 (62.1–74)	5.96	0.00	37.4 (33.9–40.9)	−7.10	0.00
	Size 30	36.8 (30.8–41.2)	−5.30	0.00	18.1 (16.4–19.8)	−36.10	0.00
	Size 40	25.9 (23.6–28.3)	−20.10	0.00	13 (11.9–14)	−71.10	0.00
*S_q_* (nm)	Size 10	88 (80.7–95.3)	10.26	0.00	46.4 (34–58.7)	−5.80	0.56
	Size 20	87.9 (80.5–95.2)	10.10	0.00	47.1 (42.6–51.6)	−1.27	0.20
	Size 30	46.7 (40.2–53.2)	−0.99	0.32	23.4 (21.3–25.5)	−24.87	0.00
	Size 40	33.7 (30.1–36.5)	−11.32	0.00	17 (15.7–18.3)	−48.77	0.00
*S_sk_*	Size 10	−0.036 (−0.149–0.078)	−0.62	0.53	−0.342 (−0.646 to −0.038)	−2.20	0.05
	Size 20	0.024 (−0.176–0.128)	0.31	0.76	0.036 (−0.126 to 0.182)	0.44	0.66
	Size 30	−0.038 (−0.148–0.072)	−0.68	0.50	−0.222 (−0.486 to 0.046)	−1.62	0.10
	Size 40	−0.43 (−0.685 to −0.175)	−3.31	0.00	−0.62 (−1.08 to −0.161)	−2.65	0.01
*S_ku_*	Size 10	4.65 (4.23–5.06)	8.20	0.00	11.86 (7.37–16.34)	3.87	0.00
	Size 20	4.77 (4.1–5.44)	5.210	0.00	4.16 (3.73–4.59)	5.340	0.00
	Size 30	4.16 (3.72–4.59)	5.310	0.00	15.61 (8.94–22.28)	3.710	0.00
	Size 40	11.4 (7.84–14.97)	4.620	0.00	29.74 (15.22–44.26)	3.610	0.00

**Table 2:** ANOVA for each respective roughness parameter with respect to prosthesis radius.

Parameter	Source of Variation	Degrees of Freedom	Sum of Squares	Mean Square	*F*-Statistic	*p*-Value
*S_a_* (nm)	Regression	1	2632	2632	37	0.001
	Error	6	426	71	–	–
	Total	7	3058	–	–	–
*S_q_* (nm)	Regression	1	4303	4303	39	0.001
	Error	6	660	110	–	–
	Total	7	4963	–	–	–
*S_sk_*	Regression	1	0.215	0.215	9.1	0.023
	Error	6	0.142	0.204	–	–
	Total	7	0.356	–	–	–
*S_sk_*	Regression	1	311	311	7.8	0.03
	Error	6	243	39	–	–
	Total	7	546	–	–	–
